# Effects of a low-protein nutritional formula with dietary counseling in older adults with chronic kidney disease stages 3–5: a randomized controlled trial

**DOI:** 10.1186/s12882-023-03423-8

**Published:** 2023-12-14

**Authors:** Wen-Ching Yang, Hui-Min Hsieh, Jun-Peng Chen, Li-Chun Liu, Cheng-Hsu Chen

**Affiliations:** 1 Department of Food and Nutrition, Taichung Veterans General Hospital, Taichung, Taiwan; 2 Biostatistics Group, Department of Medical Research, Taichung Veterans General Hospital, Taichung, Taiwan; 3 Division of Nephrology, Department of Internal Medicine, Taichung Veterans General Hospital, No. 1650, Sect. 4, Taiwan Boulevard, Taichung, Taiwan; 4 Department of Post-Baccalaureate Medicine, College of Medicine, National Chung Hsing University School of Medicine, Taichung, Taiwan; 5 Department of Life Science, Tunghai University, Taichung, Taiwan; 6 Program in Tissue Engineering and Regenerative Medicine, College of Medicine, National Chung-Hsing University, Taichung, Taiwan

**Keywords:** Chronic kidney disease, Nutritional status, Physical performance, Hand grip strength, Gait speed, Oral nutritional supplements, Aging, Low-protein diet

## Abstract

**Background:**

Although combining a low-protein diet (LPD) with oral nutritional supplements increases treatment adherence and nutritional status in patients with chronic kidney disease (CKD), the effect of this combination approach in older adults remains unclear. This study examined the impact of a 6% low-protein formula (6% LPF) with diet counseling in older adults with stage 3–5 CKD.

**Methods:**

In this three-month randomized controlled study, 66 patients (eGFR < 60 mL/min/1.73 m^2^, non-dialysis, over 65 years of age) were randomly assigned to an intervention group (LPD plus a 6% LPF) or control group (LPD alone). The 6% LPF comprised 400 kcal, 6 g of protein, eicosapentaenoic acid (EPA), docosahexaenoic acid (DHA), and various micronutrients. All data were collected at baseline and after three months, including physical performance based on hand grip strength (HGS) and gait speed, nutritional status using Mini Nutritional Assessment-Short Form (MNA-SF) scores, body composition through bioelectrical impedance analysis, and dietary intake from 24-h dietary records.

**Results:**

This study incorporated 47 participants (median age, 73; median eGFR, 36 ml/min/1.73 m^2^; intervention group: 24; control group: 23). The intervention group exhibited significant differences in HGS and gait speed, and micronutrient analysis revealed significantly higher monounsaturated fatty acids (MUFA), EPA, DHA, calcium, iron, zinc, copper, thiamine, riboflavin, niacin, B6, B12, and folic acid intake than the control group. MNA-SF scores, macronutrient intake, and body composition did not differ significantly between the two groups.

**Conclusions:**

Compared to LPD counseling alone, an LPD prescription with 6% LPF in older adults with CKD stages 3–5 helped relieve physical deterioration and increased micronutrient intake after three months.

**Trial registration:**

ClinicalTrials.gov NCT05318014 (retrospectively registered on 08/04/2022).

## Background

Chronic kidney disease (CKD) is a prevalent clinical issue that is often observed in older adults [1, 2]. Older adults with CKD may experience muscle mass shrinkage, muscle strength deterioration, decreased physical performance, and protein-energy wasting [3], contributing to mobility issues and mortality, with inadequate nutrient intake as a prominent factor [4]. Therefore, nutritional management has become essential for older adults with CKD.

Recent nutritional guidelines have suggested that a low-protein diet (LPD) can be prescribed to slow kidney function progression, improving uremia and azotemia symptoms [5–7]. However, uremic anorexia or LPD food restriction often contributes to insufficient energy and protein intake, muscle and fat loss, and reduced body weight [8]. LPDs for patients over 65 years with CKD may lower albumin levels, body weight, and body mass index (BMI) [9], mainly due to this diet’s poor caloric intake [10]. Furthermore, an LPD can lead to insufficient micronutrient intake, such as vitamins and trace elements [11], thereby exacerbating diminished physical performance risks related to other comorbidities, including weakness, frailty, and muscle spasms [12].

An LPD for older adults with CKD should be prescribed based on the individual with a thorough patient nutritional status evaluation. When addressing renal failure progression, implementing an LPD as a nutritional education guideline is challenging and should only be individually prescribed to patients with CKD after considering the patient’s adherence, age, and nutritional status [13]. Recent literature discussed the combination of dietary prescriptions and oral nutritional supplements, which support adequate dietary intake to maintain or improve body weight, body composition, and physical performance in older malnourished adults [14, 15]. Past studies have positively affected adherence to an LPD and nutritional status in patients with CKD stage 3–5 by combining low-protein nutritional formulas with dietitian dietary counseling; however, few empirical studies have focused on older adults [16, 17]. Thus, this study compared the effects of a regular LPD alone or a 6% LPF combined with a regular LPD prescription on nutrition status, physical performance, and clinical parameter changes in older adults with CKD stages 3–5.

## Methods

### Study design and participants

This study was a single-center, two-armed, open-label, parallel, randomized controlled clinical trial. Participants were recruited from August 2019 to March 2020. The Institutional Review Board of Taichung Veterans General Hospital approved this study (IRB approval number: CF19237B). All participants provided their informed consent before participating in the study. Study participants were selected and recruited by the department’s physician and dietitian investigator. The inclusion criteria were as follows: nephrology clinic outpatients over the age of 65 years who were diagnosed with stage 3–5 CKD, a mean estimated glomerular filtration rate (eGFR) < 60 ml/min/1.73 m^2^, and a non-dialysis status. The exclusion criteria were as follows: unstable conditions; leg injury; severe edema; allergies to foods such as milk, soy, or fish; use of heart rhythm devices or prosthetic limbs; relevant psychiatric disorders; and an inability to implement a 24-h dietary record. This trial was retrospectively registered at ClinicalTrials.gov NCT05318014 on August 4, 2022.

### Study treatments

Participants were allocated at a 1:1 ratio using a computer-generated random consecutive number list. Patients were divided into two groups: (1) the control group, patients received a regular LPD prescription; (2) the intervention group, patients received a regular LPD prescription with 6% LPF. All patients received an LPD prescription clinical guideline recommendation of protein intake (0.6–0.8 g/kg body weight/day) and energy requirement (30 kcal/body weight/day) [18, 19]. The LPD prescription was to assist patients in improving energy intake and implementing portion control of protein-containing foods through regular food sources. Table 1 lists the nutritional composition of 6% LPF, including a high-calorie liquid formula containing 400 kcal, 6 g of protein (6% of calories), and various nutrients (Fresubin® Renal, Germany). After face-to-face nutritional therapy during the first visit, patients were contacted by telephone to monitor their LPD prescription and adherence to 6% LPF after one and two months with the same registered dietitian during the study period. Compliance was calculated as the prescribed 6% LPF consumed after three months. Data were collected at baseline and at 3-month visits.

**Table 1:** 6% low-protein formula nutritional compositions

**Nutritional components**	**200 mL**
Energy (kcal)	400
Protein (g)	6
Carbohydrates (g)	55.2
Dietary fiber (g)	2.4
Fat (g)	17.8
Saturated fatty acids (g)	5.4
Polyunsaturated fatty acids (g)	2.9
Monounsaturated fatty acids (g)	9.4
Eicosapentaenoic acid (mg)	84
Docosahexaenoic acid (mg)	38
Sodium (mg)	136
Potassium (mg)	200
Calcium (mg)	168
Magnesium (mg)	40
Phosphorus (mg)	110
Iron (mg)	4.0
Zinc (mg)	3.6
Copper (µg)	400
Vitamin A (μg RE)	162.6
Vitamin E α-T.E (mg)	6
Vitamin D_3_ (μg)	2.0
Vitamin C (mg)	20
Thiamine (mg)	0.52
Riboflavin (mg)	0.6
Niacin (mg NE)	8.4
Vitamin B6 (mg)	1.34
Vitamin B12 (µg)	1.2
Folic acid (µg)	200

### Outcome measures

#### Nutrition status, physical activity, and body composition

The nutritional status assessment used the Mini Nutritional Assessment-Short Form (MNA-SF) [20]. Physical activity levels were measured using a Taiwanese version of the International Physical Activity Questionnaire-Short Form (IPAQ-SF) approved by the Taiwan Health Promotion Administration of the Ministry of Health and Welfare. The IPAQ-SF collected results regarding activity intensity of more than 10 min over the previous seven days and calculated the metabolic equivalent task (MET-min/week: 8 × vigorous + 4 × moderate + 3.3 × walking) by adding frequency, intensity, and duration [21, 22]. The following body composition data were collected and measured using bioelectrical impedance analysis (Tanita MC-780, Tanita Corporation of Akita Ltd., Japan): body weight (kg), BMI (kg/m^2^), body fat percentage (%), fat mass, fat-free mass, muscle mass, extracellular water, intracellular water, skeletal muscle index, resistance at 50 kHz, and phase angle.

#### Physical performance

Physical performance was measured by hand grip strength (HGS) and gait speed; HGS was measured with a TTM-YD hand dynamometer (Tsutsumi Industries, Tokyo, Japan) to identify muscle strength. Participants were placed in a sitting position with their elbow fixed at 90° and in the nondominant hand squeezed twice, with the mean results used for analysis. Gait speed was measured by physical movement via a 5-m walking test. Participants wore shoes and walked at their regular walking speed using a walking aid if necessary. The usual gait speed measured how many seconds the participant needed to walk 5 m and was recorded as m/s [23].

#### Dietary intake assessment

The 24-h dietary records using models or a food photography atlas were used to estimate portion sizes. When nutritional supplements were taken, intake frequency, dosage, and contents were recorded and incorporated into the total nutrient intake calculation. The dietitian coded and entered records obtained from an internet database created by the Food and Drug Administration regarding food and nutrition composition in Taiwan. We analyzed dietary intake, total energy intake, three macronutrient levels (carbohydrates, proteins, and lipids), fatty acid composition, saturated fatty acids (SFA), monounsaturated fatty acids (MUFA), polyunsaturated fatty acids ([PUFA]; eicosapentaenoic acid [EPA] and docosahexaenoic acid [DHA]), minerals (sodium, potassium, calcium, magnesium, phosphorus, iron, zinc, and copper), and vitamins (E, C, thiamine, riboflavin, niacin, B6, B12, and folic acid).

#### Clinical parameter measurements

Clinical parameters were collected through blood after fasting for at least 8 h, and blood urea nitrogen (BUN), creatinine, albumin, low-density lipoprotein (LDL), triglycerides, hemoglobin, and alanine aminotransferase (ALT) were recorded. The eGFR was calculated using the 4-variable Modification of Diet in Renal Disease (MDRD) study and the CKD-EPI equations [24, 25]. Laboratory analyses followed standard laboratory procedures at the Taichung Veterans General Hospital.

### Statistical analysis

The total sample size involved 47 subjects based on a power of 90% using the Wilcoxon signed-rank test and a presumed moderate effect size of 0.5 at the $\mathrm{\alpha }$ = 0.05 significance level. The sample size was calculated using G * Power version 3.1.3 (a program written by Franz Faul, Kiel University, Germany). Assuming an approximately 30% dropout rate, we considered enrolling 33 participants per group, resulting in 66 of the total participants [26, 27]. Descriptive statistics were calculated, and analysis methods were performed using the median and interquartile range (IQR) or numbers and percentages (%). Categorical variables were analyzed using the chi-square, Mann–Whitney U, and Fisher’s exact tests. Intragroup comparisons were accomplished using the Wilcoxon signed-rank test. For intergroup comparisons, data were analyzed with the Mann–Whitney U test. All data were collected, coded, verified for accuracy, and entered into a computer prior to statistical analysis with IBM® SPSS® version 22.0 (International Business Machines Corp., New York, NY, USA). Results at a *P* < 0.05 were considered statistically significant.

## Results

### Participants

**Fig. 1:**
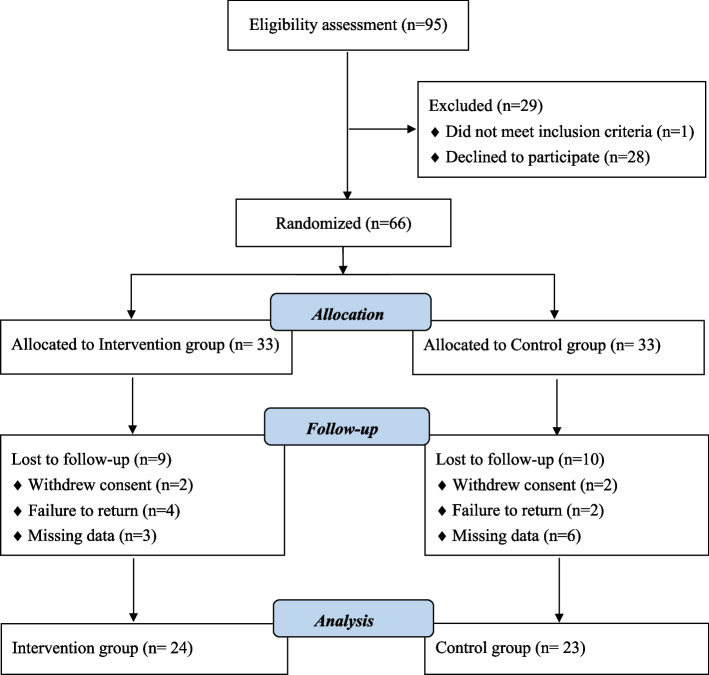
CONSORT flow diagram of participant recruitment

Participants (*n* = 95) were recruited, and 29 were excluded because they declined to participate or did not meet the inclusion criteria. Ultimately, this study included 66 participants who were randomized into two groups. After three months of follow-up, overall adherence at the study’s end revealed 47 participants the following (Fig. 1). The baseline characteristics of the two groups did not differ significantly and are presented in Table 2.

**Table 2:** Baseline data of the two older adult groups with CKD stages 3–5

	Intervention group (n = 24)		Control group (n = 23)		*P-*value
Age (years)	75	(67–81)	73	(68–78)	0.64
Male sex, n (%)	17	(70.8%)	15	(65.2%)	0.92
eGFR (mL/min/1.73 m^2^)	36	(19–45)	34	(22–50)	0.72
CKD classification, n (%)					0.27
Stage 3	17	(70.8%)	13	(56.5%)	
Stage 4	2	(8.3%)	6	(26.1%)	
Stage 5	5	(20.8%)	4	(17.4%)	
Cause of renal failure, n (%)					0.50
Hypertension	12	(50.0%)	10	(43.5%)	
Diabetes mellitus	7	(29.2%)	11	(47.8%)	
Polycystic kidney disease	2	(8.3%)	1	(4.3%)	
Other	3	(12.5%)	1	(4.3%)	
MNA-SF scores	12	(11–14)	13	(11–14)	0.26
Physical activity					
IPAQ-SF scores (MET-min/week)	953	(223–2993)	2319	(693–4986)	0.10
Body composition					
Body weight (kg)	61.0	(48.2–68.1)	67.3	(54.8–73.2)	0.06
BMI (kg/m^2)^	23.5	(20.8–25.2)	24.2	(23.0–27.4)	0.11
Body fat (%)	20.4	(17.8–23.3)	23.4	(18.8–28.7)	0.10
Fat mass (kg)	12.3	(9.9–15.0)	15.7	(10.3–20.5)	0.08
Muscle mass (kg)	44.5	(35.5–52.0)	45.8	(39.4–50.9)	0.59
Fat-free mass (kg)	47.0	(37.5–54.9)	48.9	(41.9–53.7)	0.58
Extracellular water (kg)	14.7	(12.7–16.3)	15.4	(13.9–16.0)	0.52
Intracellular water (kg)	17.9	(14.0–23.3)	19.4	(16.8–22.1)	0.46
Skeletal muscle index (kg)	7.5	(6.3–8.5)	7.8	(6.5–8.7)	0.55
Resistance at 50 kHz (Ω)	564	(504–622)	549	(502–594)	0.55
Phase angle (°)	5.5	(4.7–6.0)	5.6	(5.0–5.9)	0.65
Physical performance					
Hand grip strength (kg)	24.1	(17.0–30.6)	26.9	(19.6–31.4)	0.44
Gait speed (m/s)	0.85	(0.55–0.95)	0.91	(0.74–1.25)	0.08

Data are expressed as the median (interquartile range) or as numbers and percentages (%). Statistical differences between the two groups were determined through Chi-square, Mann–Whitney U, and Fisher's exact tests

*MNA-SF* Mini Nutritional Assessment Short-Form, *IPAQ-SF* International Physical Activity Questionnaire-Short Form, *BMI* body mass index

### Compliance with the 6% LPF

In the intervention group, the 6% LPF supplementation compliance at three months was 91.6%. A total of 33.3% of patients took 100% of the ONS, 20.8% took between 90 and 99% of the ONS, 37.5% took between 80 and 89.9% of the ONS, and 8.3% took between 70 and 79% of the ONS.

### MNA-SF, IPAQ-SF, and body composition comparisons between groups

After a three-month follow-up, the scores on the MNA-SF and IPAQ-SF did not differ significantly within the two groups or between value changes. However, while body composition data indicated increased in weight, BMI, muscle mass, and lean mass, these changes were not significant in the intervention group at the three-month follow-up. Furthermore, the two groups had no significant differences in body composition at the three-month follow-up (Table 3).

**Table 3:** MNA-SF, physical activity, body composition, and physical performance outcomes

	Intervention group (*n* = 24)						Control group (*n* = 23)						*P-*value^b^
	Baseline		3 months		Change in value (3 months-Baseline)^a^		Baseline		3 months		Change in value (3 months-Baseline)^a^		
MNA-SF scores	12	(11–14)	13	(11–14)	0	(0–2)	13	(11–14)	13	(13–14)	0	(0–1)	0.95
Physical activity													
IPAQ-SF scores (MET-min/week)	953	(223–2993)	1040	(473–2993)	0	(0–256)	2319	(693–4986)	1446	(882–4452)	0	(-693–0)	0.084
Body composition													
Body weight (kg)	61.0	(48.2–68.1)	61.0	(50.1–68.1)	0.4	(-1.5–1.6)	67.3	(54.8–73.2)	67.2	(57.1–72.9)	-0.3	(-1.0–0.1)	0.35
BMI (kg/m^2)^	23.5	(20.8–25.2)	23.6	(20.7–25.4)	0.1	(-0.2–0.9)	24.2	(23.0–27.4)	24.6	(23.0–27.5)	0.0	(-0.3–0.7)	0.51
Body fat (%)	20.4	(17.8–23.3)	17.9	(13.9–25.3)	-1.6	(-4.5–1.2)	23.4	(18.8–28.7)	25.1	(21.1–30.5)	-0.2	(-1.6–2.0)	0.10
Fat mass (kg)	12.3	(9.9–15.0)	10.9	(6.3–16.2)	-0.8	(-2.7–0.9)	15.7	(10.3–20.5)	15.6	(11.6–19.5)	-0.1	(-1.0–1.5)	0.16
Muscle mass (kg)	44.5	(35.5–52.0)	45.8	(35.7–50.9)	0.2	(-0.7–2.4)	45.8	(39.4–50.9)	43.9	(39.4–50.6)	0.0	(-1.3–0.5)	0.19
Fat-free mass (kg)	47.0	(37.5–54.9)	48.3	(37.8–53.6)	0.3	(-0.8–2.6)	48.9	(41.9–53.7)	46.3	(41.9–53.4)	0.0	(-1.4–0.6)	0.19
Extracellular water (kg)	14.7	(12.7–16.3)	14.9	(12.9–16.2)	0.0	(-0.4–0.6)	15.4	(13.9–16.0)	15.4	(13.9–16.1)	0.0	(-0.3–0.2)	0.59
Intracellular water (kg)	17.9	(14.0–23.3)	18.9	(15.1–23.2)	0.3	(-0.4–1.3)	19.4	(16.8–22.1)	19.1	(16.7–21.8)	0.2	(-0.8–1.3)	0.47
Skeletal muscle index (kg)	7.5	(6.3–8.5)	7.5	(6.3–8.7)	0.0	(-0.2–0.2)	7.8	(6.5–8.7)	7.6	(6.7–9.0)	0.0	(-0.2–0.3)	0.83
Resistance at 50 kHz (Ω)	564	(504–622)	552	(472–615)	-8	(-36–12)	549	(502–594)	527	(486–598)	2	(-30–11)	0.58
Phase angle (°)	5.5	(4.7–6.0)	5.5	(5.1–6.9)	0.0	(-0.5–0.5)	5.6	(5.0–5.9)	5.3	(5.0–5.7)	-0.1	(-0.6–0.1)	0.18
Physical performance													
Hand grip strength (kg)	24.1	(17.0–30.6)	25.4	(17.3–33.3)	0.8	(-0.8–3)	26.9	(19.6–31.4)	25.6	(18.0–30.3)	-1.2	(-2.6–0.1)^†^	0.004**
Gait speed (m/s)	0.85	(0.55–0.95)	0.88	(0.59–1.17)	0.07	(-0.02–0.22)^†^	0.91	(0.74–1.25)	0.80	(0.64–1.11)	-0.03	(-0.19–0.06)	0.009**

Data are expressed as the median (interquartile range)

*MNA-SF* Mini Nutritional Assessment Short-Form, *IPAQ-SF* International Physical Activity Questionnaire-Short Form, *BMI* body mass index

^a^Groups from baseline to three months were compared through the Wilcoxon signed-rank test (†*P* < 0.05)

^b^Value change differences of the two groups as determined by the Mann–Whitney U test (*P < 0.05 and ** *P* < 0.01)

### Physical performance effects

**Fig. 2:**
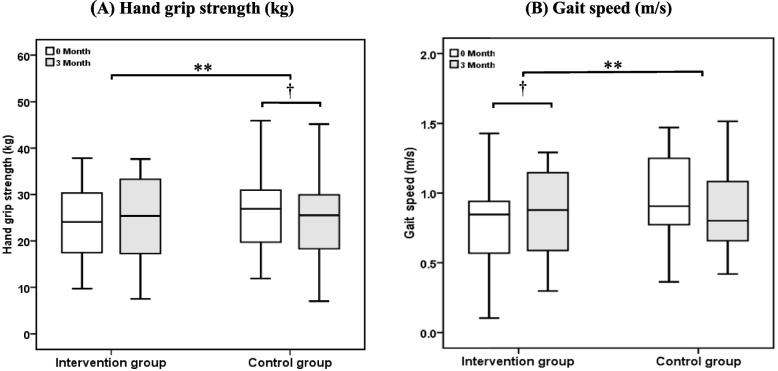
The box plot displays the physical performance parameters from a three-month treatment follow-up. **A** Hand grip strength (kg). **B** Gait speed (m/s). Data are expressed as the median (interquartile range). Comparisons within groups from baseline to three months were performed using the Wilcoxon signed-rank test (†*P* < 0.05). Value change differences between intervention and control groups as determined by the Mann–Whitney U test (*P < 0.05, ** *P* < 0.01)

HGS (kg) decreased in the control group from baseline to three months, with a change of -1.2 kg (IQR, -2.6 to 0.1; *P* = 0.022). In contrast, the intervention group did not exhibit any significant differences. Furthermore, gait speed (m/s) slightly increased in the intervention group, with a change of 0.07 m/s (IQR, -0.02 to 0.22; *P* = 0.048) (Table 3). There was a significant difference in value changes for HGS (kg) and gait speed (m/s) when comparing the intervention group to the control group (*P* = 0.004 and *P* = 0.009, respectively) (Fig. 2).

### Results from 24-h diet records

Baseline calcium intake was higher in the control group than in the intervention group, without any other significant differences (data not shown). In terms of macronutrient intake, there was no significant difference between the two groups. Regarding fatty acid components, MUFA and EPA intake significantly increased in the intervention group, and the MUFA, EPA, and DHA intake changes heightened considerably between the two groups. For mineral intake, calcium, iron, zinc, and copper all significantly increased within the intervention group. Additionally, the intervention group’s change in intake increased notably more than that of the control group. For vitamin component intake, including thiamine, riboflavin, niacin, vitamin B6, vitamin B12, and folate, there were significant increases within the intervention group. Similarly, value changes in the intervention group showed a significant increase in vitamin component intake compared with the control group (Table 4).

**Table 4:** Macronutrients, Fatty acids, and micronutrients outcomes assessment with 24-h dietary records

	Intervention group (*n* = 24)			Control group (*n* = 23)			*P*-value^b^
	Baseline	3 months	Change in value (3 months-Baseline)^a^	Baseline	3 months	Change in value (3 months-Baseline)^a^	
**Macronutrients**							
Energy (kcal)	1567 (1288–1837)	1632 (1472–1852)	137 (-67–200)	1521 (1284–1704)	1512 (1273–1700)	6 (-175–175)	0.20
Energy (kcal/kg body weight)	26.0 (22.1–35.0)	29.4 (23.3–34.7)	1.8 (-1.6–4.4)	23.2 (18.6–27.4)	22.5 (20.7–26.1)	0.1 (-2.8–2.4)	0.19
Protein intake (g)	52.5 (36.8–68.7)	50.5 (42.8–56.6)	-3.7 (-14.7–5.3)	46.7 (41.6–55.6)	48.2 (43.7–52.9)	2.1 (-7.4–9.9)	0.37
Protein intake (g/kg body weight)	0.88 (0.66–1.21)	0.85 (0.65–1.05)	-0.07 (-0.23–0.09)	0.73 (0.55–0.93)	0.75 (0.62–0.86)	0.05 (-0.15–0.14)	0.41
Carbohydrate intake (g)	213 (184–249)	222 (179–257)	12 (-17–38)	194 (165–239)	213 (168–241)	2 (-36–41)	0.87
Fat intake (g)	61 (46–70)	68 (54–74)	6 (-5–23)	51 (43–70)	50 (45–66)	-1 (-11–9)	0.08
Dietary fiber (g)	11.4 (8.6–15.1)	12.2 (7.6–11.8)	2.1 (-3.3–4.7)	12.5 (7.1–15.1)	10.6 (9.3–15.6)	-0.6 (-4.6–3.4)	0.36
**Fatty acids composition**							
SFA (g)	15.9 (10.2–18.2)	16.7 (13.6–21.2)	4.0 (-2.5–8.1)	11.9 (9.2–16.1)	12.8 (9.3–16.3)	-0.3 (-4.2–3.1)	0.17
PUFA (g)	19.1 (15.7–25.7)	23.6 (16.1–26.5)	1.8 (-3.9–8.5)	22.4 (17.1–32.3)	20.8 (16.4–23.0)	-2.1 (-12.2–3.1)	0.09
MUFA (g)	17.6 (11.9–21.0)	24.8 (20.0–27.8)	7.9 (2.5–11.7)^§^	13.1 (10.8–21.1)	12.9 (10.5–18.4)	-0.9 (-5.3–1.2)	< 0.001**
EPA (mg)	17.9 (4.5–60.9)	109.4 (86.7–130.3)	83.0 (47.0–105.9)^§^	4.5 (1.0–86.6)	4.6 (1.4–12.6)	-0.4 (-81.9–3.6)	< 0.001**
DHA (mg)	88.4 (46.7–160.4)	119.8 (106.3–209.4)	48.7 (-74.9–145.3)	62.1 (34.4–372.6)	41.9 (27.8–73.7)	-20.4 (-149.4–1.6)^§^	0.009**
**Micronutrients**							
Sodium (mg)	1138 (470–1966)	1132 (454–1615)	19 (-759–451)	1121 (668–1807)	1258 (656–1831)	15 (-554–601)	0.70
Potassium (mg)	1463 (1004–2017)	1403 (934–2335)	89 (-522–668)	1336 (963–1972)	1346 (1024–2087)	170 (-521–589)	0.88
Calcium (mg)	206 (156–299)	447 (319–759)	198 (113–384)^§^	421 (225–564)	392 (249–588)	-47 (-332–317)	0.014*
Magnesium (mg)	143 (106–202)	152 (129–218)	33 (-50–80)	133 (110–208)	149 (107–201)	12 (-34–69)	0.88
Phosphorus (mg)	593 (466–777)	647 (499–732)	-4 (-165–189)	595 (498–769)	592 (490–773)	54 (-295–109)	0.57
Iron (mg)	6.0 (4.7–8.8)	10.1 (8–13.7)	4.3 (2.0–6.3)^§^	6.5 (4.6–10.4)	6.9 (3.2–9.8)	-1.2 (-2.5–1.8)	< 0.001**
Zinc (mg)	6.8 (5.0–8.2)	9.0 (8.3–9.6)	2.3 (0.6–3.4)^§^	5.9 (4.9–7.4)	6.2 (5.5–6.7)	0.0 (-1.2–1.3)	0.001**
Copper (μg)	42.9 (10.6–65.2)	423.5 (409.2–447.4)	397.1 (356.0–415.1)^§^	39.3 (4.2–56.4)	13.6 (5.8–41.5)	-5.1 (-31.7–8.8)	< 0.001**
Vitamin E α-T.E (mg)	27.0 (16.9–34.6)	31.8 (20.3–36.8)	5.2 (-6.5–15.5)	31.6 (21.6–40.2)	26.9 (22.8–29.6)	-0.1 (-10.9–7.8)	0.11
Vitamin C (mg)	68 (53–149)	91 (57–195)	24 (-35–114)	101 (43–166)	108 (63–215)	-13 (-71–109)	0.54
Thiamine (mg)	0.8 (0.6–0.9)	1.2 (1–1.3)	0.4 (0.2–0.6)^§^	0.8 (0.5–1.1)	0.8 (0.6–1.2)	0.1 (-0.3–0.5)	0.037*
Riboflavin (mg)	0.7 (0.5–1.2)	1.3 (1.1–1.5)	0.5 (0.2–0.8)^§^	0.9 (0.5–1.2)	0.8 (0.5–1.2)	0.1 (-0.3–0.3)	< 0.011**
Niacin (mg NE)	8.0 (5.6–11.3)	15.7 (13.2–17.1)	7.5 (3.3–9.7)^§^	8.5 (5.1–12.1)	8.1 (5.5–12.6)	0.3 (-4.5–2.8)	< 0.001**
Vitamin B_6_ (mg)	1.0 (0.9–1.4)	2.3 (2–2.6)	1.2 (0.9–1.4)^§^	0.9 (0.7–1.5)	1.0 (0.8–1.3)	0.0 (-0.1–0.2)	< 0.001**
Vitamin B_12_ (μg)	1.8 (1.2–2.3)	2.7 (2.2–3.7)	1.1 (0.2–2.1)^§^	1.2 (0.8–2.4)	1.5 (0.6–1.8)	-0.4 (-1.6–0.9)	0.006**
Folate (μg)	150 (91–221)	313 (267–373)	162 (75–233)^§^	125 (89–177)	107 (76–165)	-9 (-70–75)	< 0.001**

Data are expressed as the median (interquartile range)

*SFA* saturated fatty acids, *PUFA* polyunsaturated fatty acids, *MUFA* monounsaturated fatty acids, *EPA* eicosapentaenoic acid, *DHA* docosahexaenoic acid

^a^Groups from baseline to 3 months were compared through the Wilcoxon signed-rank test (^§^*P* < 0.01)

^b^Value change differences between the two groups as determined by the Mann–Whitney U test (*P < 0.05 and ** *P* < 0.01)

### Clinical parameter outcomes

Baseline clinical values did not differ significantly between groups. From baseline to three months, BUN induced a -2 mg/dl value change (IQR, -6 to -1; *P* = 0.03) in the intervention group, whereas no changes were observed in the control group (*P* = 0.80). There were no statistically significant changes in eGFR and other clinical parameters, and no significant differences were observed in the groups’ value shifts (Table 5).

**Table 5:** Clinical parameter outcomes from the two groups in older adults with stage 3–5 CKD

	Intervention group (*n* = 24)			Control group (*n* = 23)			*P*- value^*b*^
	Baseline	3 months	Change in value (3 months-Baseline)^a^	Baseline	3 months	Change in value (3 months-Baseline)^a^	
BUN (mg/dl)	31 (25–43)	29 (22–43)	-2 (-6- -1)^†^	31 (23–46)	26 (19–53)	-2 (-5–1)	0.75
eGFR (mL/min per/1.73 m^2^)							
MDRD	36 (19–45)	38 (16–44)	-1 (-3–3)	34 (22–50)	31 (19–50)	-1 (-4–1)	0.51
CKD-EPI (2009)	31 (16–42)	34 (14–39)	-1 (-3–2)	32 (20–46)	28 (17–46)	0 (-4–1)	0.78
CKD-EPI (2021)	34 (18–46)	37 (15–43)	-1 (-3–3)	34 (22–50)	30 (19–50)	-4 (-1–1)	0.78
Creatinine (mg/dl)	1.7 (1.6–3.4)	1.7 (1.5–3.7)	0.0 (-0.1–0.3)	1.9 (1.4–3.0)	1.9 (1.4–3.4)	0.1 (-0.1–0.3)	0.35
LDL (mg/dL)	89 (77–124)	94 (73–134)	-2 (-14–13)	88 (72–115)	82 (70–114)	-2 (-17–10)	0.82
Triglyceride (mg/dl)	108 (83–164)	120 (77–194)	7 (-12–32)	111 (74–146)	119 (79–143)	0 (-20–22)	0.20
Albumin (g/dl)	4.2 (4.1–4.4)	4.2 (4.1–4.3)	0.0 (-0.2–0.1)	4.2 (3.9–4.4)	4.2 (3.8–4.4)	0.0 (-0.2–0.1)	0.92
Hemoglobin (g/dl)	11.8 (10.2–13.7)	12.2 (10.5–13.7)	0.0 (-0.7–0.7)	12.3 (9.8–13.7)	12.1 (9.6–14.0)	-0.4 (-0.9–0.3)	0.30
ALT (U/L)	18.5 (12.3–21.0)	17.5 (11.3–21.0)	-1.0 (-5.0–2.8)	18.0 (14.0–24.0)	17.0 (14.0–31.8)	0.0 (-3.0–3.5)	0.43

Data are expressed as the median (interquartile range)

*BUN *blood urea nitrogen, *eGFR*, estimated glomerular filtration rate, *MDRD *modification of diet in renal disease, *LDL *low-density lipoprotein, *ALT *alanine aminotransferase

^a^Groups were compared through the Wilcoxon signed-rank test (^†^*P* < 0.05)

^b^Value change differences between the two groups as determined by the Mann–Whitney U test

## Discussion

This study is the first to explore oral nutritional supplement effects in an older individual in stage 3–5 CKD at the 3-month follow-up period. We discovered that maintaining nutritional status consists of the MNA-SF, physical activity and body composition in both groups. A recent systematic review indicated that dialysis treatment involving a mix of various macronutrients for up to 12 months significantly increased lean body mass [28]. In a previous single-arm study, 35 patients with stage 3b-5 CKD adhering to a dietitian-guided LPD and a renal-specific formula noted considerable improvements in body weight and grip strength over six months [29]. In contrast with past studies, our findings showed that an LPD combined with a 6% LPF prompted a slight increase in body weight, muscle mass, and fat-free mass. Although the data were not significantly different between groups over three months, future long-term studies may be able to observe such differences.

In a previous study, an LPD of approximately 85% for patients with CKD stages 3–5 offered an insufficient daily calorie intake despite receiving standard routine diet counseling, while also associated with a slight decrease in gait speed [30]. These study data suggest that with an LPD prescription plus a 6% LPF, an increase in HGS and gait speed was observed compared to the LPD prescription alone. This result seems to imply that an LPD prescription plus a 6% LPF was a positive steady delay in physical function.

This study's results indicated that an LPD plus a 6% LPF provided no changes in energy and protein intake while increasing fatty acid and specific micronutrient intake during the 3-month follow-up period. Outcomes could be attributed to the two groups following the target calorie and protein intake under a dietitian’s guidance. The intervention group used oral nutritional supplements (6% LPF) to replace high-protein foods and achieve target energy intake and protein control. The control group used natural food during implementation. In a previous survey study, protein intake restrictions required energy supplements to avoid malnutrition and protein-energy wasting, potentially facilitating the use of protein-free products or other options as their primary energy source [31]. Furthermore, LPDs often lack variety, increasing the chances that a patient’s micronutrient intake will be lower [12]. This may be consistent with our finding, resulting in a decline in the intake of fatty acids and multivitamins estimated under the control of protein intake. However, maintaining a low-protein diet while consuming sufficient calories and nutrients may not be achieved through fatty acid or micronutrient supplementation alone.

Recent reports found that n-3 PUFA supplementation through a fish oil source enhanced muscle protein synthesis rates in older adults, improving muscle strength and boosting physical performance [32, 33]. Vitamin B and certain minerals are closely related to physical function [34, 35], and additional reports have suggested that administering multiple nutrients may be more effective than single-nutrient supplementation to prevent age-related muscle mass and strength attenuation [36–38]. However, it is unclear which types of nutrients affect muscle strength and gait speed in older adults with CKD stages 3–5. An LPD regimen with a 6% LPF prescription may be applied favorably in physical performance.

Regarding clinical parameters, this study also concluded that BUN was significantly reduced in the intervention group over three months. Previous randomized controlled trials have suggested that low-protein formula supplements could improve LPD adherence and kidney function outcomes [16, 17]*.* One case‒control observational study demonstrated that nutritional consulting was helpful for phosphorus and BUN clinical indications compared to no nutritional consulting [39]. Although kidney function results were inconsistent, a renal dietitian may have advised all participants. The results of these previous studies provide potential reasons why a 6% LPF does not significantly change renal function compared to a control group in older adults with CKD stages 3–5.

This study has several limitations. First, this study was conducted in a single center; therefore, the generalizability of the results may be poor. Second, concerning dietary intake, although previous studies of homebound older adults have proven reliable using a 24-h dietary record for energy intake, there may be a validity bias when describing information [40]. Third, the study design was open-label, and outcome assessments may have been biased in both groups. The results measuring physical activity (IPAQ-SF), which may influence bias, and no significant change between the two groups. Therefore, physical performance was chosen as the objective measure to mitigate outcome effects. Additionally, three months may be too short to identify the full impact of the outcome. Finally, all participants were adults over 65 and could not be extrapolated to other age groups, excluding other age group populations.

## Conclusions

This study established that an LPD prescription with a 6% LPF can delay physical performance deterioration and increase micronutrient intake in three months compared to LPD education alone in older adults with CKD stages 3–5. Future studies should incorporate other age groups and a longer duration to validate these findings.

## Data Availability

The datasets analyzed during this study are available through the corresponding author upon reasonable request.

## Abbreviations

ALT: Alanine aminotransferase
BMI: Body mass index
BUN: Blood urea nitrogen
CKD: Chronic kidney disease
DHA: Docosahexaenoic acid
eGFR: Estimated glomerular filtration rate
EPA: Eicosapentaenoic acid
IQR: Interquartile range
IPAQ-SF: International Physical Activity Questionnaire-Short Form
LDL: Low-density lipoprotein
LPD: Low protein diet
6% LPF: 6% Low-protein formula
HGS: Hand grip strength
MNA-SF: Mini Nutritional Assessment Short-Form
MUFA: Monounsaturated fatty acids
PUFA: Polyunsaturated fatty acids
SFA: Saturated fatty acids
